# Cost-effectiveness analysis of tislelizumab, nivolumab and docetaxel as second- and third-line for advanced or metastatic non-small cell lung cancer in China

**DOI:** 10.3389/fphar.2022.880280

**Published:** 2022-08-25

**Authors:** Dongchu Zhou, Xia Luo, Zhen Zhou, Xiaohui Zeng, Xiaomin Wan, Chongqing Tan, Qiao Liu

**Affiliations:** ^1^ Department of Pharmacy, The Second Xiangya Hospital of Central South University, Changsha, China; ^2^ Menzies Institute for Medical Research, University of Tasmania, Hobart, TAS, Australia; ^3^ Department of Nuclear Medicine/PET Image Center, The Second Xiangya Hospital of Central South University, Changsha, China

**Keywords:** cost-effectiveness, NSCLC, tislelizumab, nivoluma, docetaxel, China

## Abstract

**Objective:** Domestic PD-1inhibitor tislelizumab has emerged as a promising treatment for Chinese patients with driver-negative advanced or metastatic non-small cell lung cancer (NSCLC). The purpose of our study to evaluate whether tislelizumab is cost-effective as a second- or third-line treatment for this population compared with docetaxel (conventional chemotherapy) and nivolumab (imported PD-1inhibitor), from the perspective of the Chinese healthcare system.

**Material and Methods:** A Markov model with a 3-week Markov cycle and a 30-year time horizon was built to compare the cost-effectiveness of second- or third-line tislelizumab versus docetaxel and nivolumab. Transition probabilities, including disease progression, survival, and adverse events (AEs)-related treatment discontinuation event, were estimated from the clinical trials. Costs and health utilities were collected from local hospitals, public database and published literature.

**Results:** Compared with docetaxel, tislelizumab provided an additional 0.33 quality-adjusted life-years (QALYs) (1.37 vs. 1.04 QALYs) at an incremental cost of $9,286 ($23,646 vs. $14,360) for Chinese patients with driver-negative advanced or metastatic NSCLC, resulting in an incremental cost-effectiveness ratio (ICER) of $27,959/QALY under the WTP threshold of $35,663/QALY used in the model. Compared with nivolumab, tislelizumab was associated with a lower cost ($23,646 vs. $59,447) and higher QALYs (1.37 vs. 1.20 QALYs), resulting in its dominance of nivolumab.

**Conclusion:** From the perspective of the Chinese healthcare system, domestic PD-1inhibitor tislelizumab immunotherapy represents a cost-effective treatment strategy compared with conventional docetaxel chemotherapy and imported PD-1inhibitor nivolumab immunotherapy in the treatment of driver-negative advanced or metastatic NSCLC beyond the first-line setting. In the era of “Universal Medical Insurance System”, the rational use of domestic anticancer drugs guided by cost-benefit evidence would be an effective means to balance the limited expenditure of medical insurance fund and the growing demand for cancer treatments.

## Introduction

In 2020, China reported 816,000 new lung cancer cases which ranked first in the world (Cao et al., 2021; Sung et al., 2021). Non-small cell lung cancer (NSCLC) accounted for about 85% of these cases (Tian et al., 2022). Up to 46% of NSCLC cases had an advanced or metastatic disease (Chen et al., 2014), and a substantial proportion of patients had no genetic aberrations, resulting in their ineligibility for promising targeted therapy (Grant et al., 2021). For driver-negative advanced or metastatic NSCLC patients (defined as advanced or metastatic NSCLC patients without known sensitizing EGFR mutations or ALK rearrangements), who progressed after prior platinum-based chemotherapy, anti-programmed cell death protein 1/programmed cell death ligand 1 (PD-1/L1) immunotherapy becomes the mainstay of the standard-of-care (Guidelines Working Committee of Chinese society of Clinical Oncology, 2021). As of now, the Chinese National Medical Products Administration (NMPA) approved 3 anti PD-1/L1 therapies (nivolumab, pembrolizumab, atezolizumab) for the management of driver-negative advanced or metastatic NSCLC, given their superior efficacy in prolonging survival over traditional chemotherapies (Herbst et al., 2016; Rittmeyer et al., 2017; Wu et al., 2019). However, the prohibitive costs of these imported drugs (about $84,000 per year) substantially limit their widespread uses in China, where the per capita gross domestic product (GDP) is only $10,000 (National Bureau Of Statistics Of China, 2021).

To improve patients’ accessibility to PD-1/L1 inhibitors, the Chinese government has been committed to the development of domestic PD-1/L1 inhibitors in recent years (Central People’s Government of the People’s Republic of China, 2021a). Tislelizumab is the first domestic anti-PD-1 antibody that shows good efficacy in the second- and third-line treatments for advanced or metastatic NSCLC (Zhou et al., 2021). The ongoing RATIONALE 303 is an open-label, randomized phase three trial of tislelizumab versus docetaxel in NSCLC subjects who have progressed after the prior platinum-containing treatment; (ClinicalTrials.gov Identifier: NCT03358875) (Zhou et al., 2021). The research team recently reported that tislelizumab significantly improved overall survival (OS) and progression-free survival (PFS) in the study population compared to docetaxel (Zhou et al., 2021). In addition, tislelizumab showed a good safety profile of which most adverse events (AEs) were tolerable and manageable. Also, tislelizumab users reported fewer grade III/IV adverse events (AEs) than those treated with docetaxel (Zhou et al., 2021). According to this, the Chinese society of clinical oncology (CSCO) Guidelines recommend tislelizumab used as a second-line treatment option for patients with driver-negative advanced or metastatic NSCLC (Guidelines Working Committee of Chinese society of Clinical Oncology, 2021).

The efficacy of domestic PD-1inhibitors must be weighed against its economic consequences. Whether domestic PD-1inhibitor tislelizumab provides an additional clinical value at a justifiable cost compared to commonly used clinical therapies, such as imported PD-1inhibitor nivolumab immunotherapy and conventional docetaxel chemotherapy, remains to be determined. Therefore, we conducted this study to assess the cost-effectiveness of tislelizumab versus docetaxel and nivolumab as a second- or third-line treatment for advanced or metastatic NSCLC from the perspective of the Chinese healthcare system.

## Methods

### Overview

We designed a cost-effectiveness model to compare the use of tislelizumab (domestic PD-1inhibitor), docetaxel (chemotherapy drug), and nivolumab (imported PD-1inhibitor) in the treatment of driver-negative advanced or metastatic NSCLC beyond the first-line setting from the perspective of Chinese health care system. TreeAge Pro Healthcare software (version 2021, https://www.treeage.com/) and R software (version 4.0.4, http://www.r-project.org) were used to construct and analyze this model. This economic evaluation used non-individual patient data and was therefore deemed exempt from the approval of Chinese ethics review committee. Our study followed the China Guidelines for Pharmacoeconomic Evaluation (2020) (Chinese Pharmaceutical Association, 2020).

### Model construction

**FIGURE 1:**
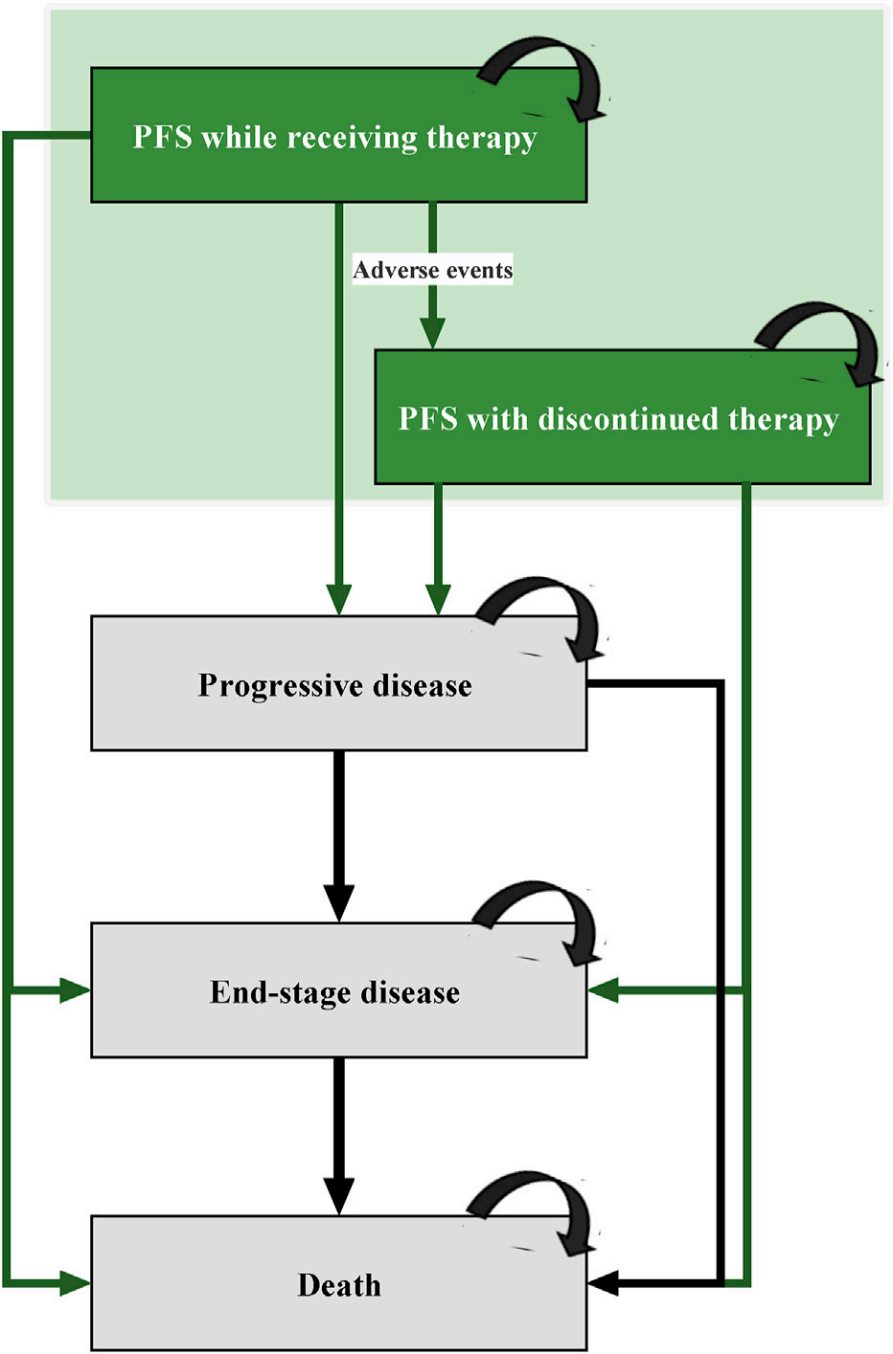
Diagram of Markov Model. PFS, progression-free survival.

This analysis based on a Markov model was characterized by four main health states: progression-free survival (PFS), progressive disease (PD), end-stage disease, and death (Figure 1). Model patients mirrored the subjects recruited in the RATIONALE 303 clinical trial, who had driver-negative advanced or metastatic NSCLC and progressed after previous platinum-based chemotherapy. All patients began in PFS health state and were treated with second- or third-line tislelizumab, docetaxel, and nivolumab. Considering that patients may discontinue these treatments due to intolerable toxicity before experiencing disease progression (Lu et al., 2021a; Zhou et al., 2021), two sub PFS health states (PFS health state while receiving therapy and PFS health state with discontinued therapy) were constructed to reflect the real-world practice. Patients with disease progression during tislelizumab, docetaxel, or nivolumab treatments would transfer to the PD health state, in which certain patients were proceeded to third- or further-line treatment with anlotinib (Han et al., 2018; Guidelines Working Committee of Chinese society of Clinical Oncology, 2021). After progressed on anlotinib treatment, patients ultimately entered to the end-stage disease health state and were provided with palliative care before death (Guidelines Working Committee of Chinese society of Clinical Oncology, 2021). In addition, according to the CSCO Guidelines for NSCLC, the best supportive care (BSC) should be supplemented in patients receiving cancer treatments (Guidelines Working Committee of Chinese society of Clinical Oncology, 2021). Supplementary Table S1 provides the dosage and administration information for each treatment regimen used in the model.

Markov cohort analysis with a 3-week Markov cycle and a 30-year time horizon was performed to compute the incremental cost-effectiveness ratio (ICER) between competing treatment options, which reflected the incremental healthcare cost consumed for each additional effectiveness [measured by quality-adjusted life-year (QALY)]. The cost-effectiveness of one regimen relative to another was determined by comparing these ICERs with the willingness-to-pay (WTP) threshold of $35,663 per QALY (defined as three times of China’s per capita GDP in 2021) (Chinese Pharmaceutical Association, 2020; National Bureau Of Statistics Of China, 2021). This study reported costs in 2021 USD (1 USD was equivalent to 6.4512 CNY) and discounted both costs and effectiveness at an annual rate of 5% (Chinese Pharmaceutical Association, 2020).

### Transition probabilities

Transition probabilities between Markov health states were estimated using the method described in our previous studies (Liu et al., 2021a; Liu et al., 2021b). Firstly, using the GetData Graph Digitizer software (version 2.26; http://www.getdata-graphdigitizer.com/index.php), we digitized the survival data for the second- or third-line tislelizumab/docetaxel and third- or further-line anlotinib from the Kaplan-Meier (KM) curves reported in their representative clinical trials (Han et al., 2018; Zhou et al., 2021). Secondly, according to goodness-of-fit test using Akaike information criterion (AIC) and Bayesian information criterion (BIC), we chose the log-logistic distribution that provided the best fit to these recreated individual patient-level data for survival fitting (Supplementary Figure S1 and Supplementary Table S2). Thirdly, the log-logistic survival curves for second- or third-line nivolumab were derived by using the HRs of nivolumab relative to tislelizumab, which were generated by a network meta-analysis implemented in R software due to the lack of head-to-head clinical trials for a direct comparison. Fourthly, the log-logistic theta (θ) and kappa (κ) parameters were used to calculate transition probabilities between four main Markov health states (16.17). Finally, the transition probability between the two PFS substates was calculated using data of AEs-related treatment discontinuation observed in clinical trials (Han et al., 2018; Zhou et al., 2021) (Supplementary Table S3). All model inputs for transition probabilities estimation are summarized in Table 1.

**TABLE 1:** Model inputs.

Variable	Baseline value	Range	Distribution	Source
**Survival**				
OS for second- or third-line tislelizumab	Log-logistic:θ = 0.00927; κ = 1.46070	Fixed in DSA	Fixed in PSA	Estimated^a^
PFS for second- or third-line tislelizumab	Log-logistic:θ = 0.09158; κ = 1.28272	Fixed in DSA	Fixed in PSA	Estimated^a^
OS for second- or third-line docetaxel	Log-logistic:θ = 0.01092; κ = 1.61683	Fixed in DSA	Fixed in PSA	Estimated^a^
PFS for second- or third-line docetaxel	Log-logistic:θ = 0.05747; κ = 1.96409	Fixed in DSA	Fixed in PSA	Estimated^a^
OS for third- or furth-line anlotinib	Log-logistic:θ = 0.01184; κ = 1.69854	Fixed in DSA	Fixed in PSA	Estimated^a^
PFS for third- or furth-line anlotinib	Log-logistic:θ = 0.01411; κ = 2.18608	Fixed in DSA	Fixed in PSA	Estimated^a^
HR_OS_ of second- or third-line nivolumab vs. tislelizumab	1.170	0.509–2.683	Normal	Estimated^b^
HR_OS_ of second- or third-line nivolumab vs. tislelizumab (male subgroups)	1.342	0.469–2.839	Normal	Estimated^a^
HR_OS_ of second- or third-line nivolumab vs. tislelizumab (female subgroups)	0.740	0.370–1.484	Normal	Estimated^a^
HR_PFS_ of second- or third-line nivolumab vs. tislelizumab	1.235	0.540–2.844	Normal	Estimated^b^
1-Cycle probability of tislelizumab treatment discontinuation due to AEs	0.004499	0.002249–0.006748	Beta	Estimated^c^
1-Cycle probability of docetaxel treatment discontinuation due to AEs	0.007759	0.003880–0.011639	Beta	Estimated^c^
1-Cycle probability of nivolumab treatment discontinuation due to AEs	0.003408	0.001704–0.005112	Beta	Estimated^c^
**Costs (US$)**				
Tslelizumab per 200 mg	675.84	337.92–1013.76	Gamma	Local charge
Docetaxel per 75 mg	39.53	19.76–59.29	Gamma	Local charge
Nivolumab per 3 mg	43.02	21.51–64.52	Gamma	Local charge
Anlotinib per 168 mg	665.92	332.96–998.88	Gamma	Local charge
Routine follow-up per cycle	55.60	27.80–83.40	Gamma	Liu Q et al.
BSC per cycle	337.50	168.75–506.25	Gamma	Liu Q et al.
Palliative care per cycle	2627.80	1313.90–3941.70	Gamma	Liu Q et al.
AEs cost for second- or third-line tislelizumab	89.36	44.68–134.04	Gamma	Estimated^d^
AEs cost for second- or third-line docetaxel	1212.99	606.50–1819.49	Gamma	Estimated^d^
AEs cost for second- or third-line nivolumab	13.39	6.70–20.09	Gamma	Estimated^d^
**Utilities**				
PFS health state	0.856	0.718–0.994	Beta	Shen Y et al.
PD health state	0.768	0.595–0.941	Beta	Shen Y et al.
End-stage disease health state	0.703	0.545–0.861	Beta	Shen Y et al.
AEs disutility for second- or third-line tislelizumab	0.002	0.001–0.003	Beta	Estimated^d^
AEs disutility for second- or third-line docetaxel	0.061	0.030–0.091	Beta	Estimated^d^
AEs disutility for second- or third-line nivolumab	0.002	0.001–0.003	Beta	Estimated^d^
**Other**				
Discount rate (%)	5	0–8	Fixed in PSA	Guidelines
Patient weight (kg)	65	32.5–97.5	Normal	Lu S et al.
Body surface area (m^2^)	1.72	0.86–2.58	Normal	Lu S et al.
Proportion of subsequent anticancer therapy in tislelizumab group	49.7%	24.9–74.6%	Beta	RATIONALE 303 trial
Proportion of subsequent anticancer therapy in docetaxel group	62.6%	31.3–93.9%	Beta	RATIONALE 303 trial
Proportion of subsequent anticancer therapy in nivolumab group	45.0%	22.5–67.5%	Beta	CheckMate 078 trial

a Estimated by the survival fitting implemented in R software.

b Estimated by the network meta-analysis implemented in R software.

c Estimated in Supplemental Table S3.

d Estimated in Supplemental Table S4.

DSA, deterministic sensitivity analysis; PSA, probabilistic sensitivity analyses; OS, overall survival; PFS, progression-free survival; HR, hazard ratio; BSC, best supportive care; AEs: adverse events; PD, progressive disease.

### Costs and utilities estimates

We collected data of costs from the perspective of the Chinese healthcare system and incorporated the costs of second- and further-line drugs, AE management and general cancer management (including routine follow-up, BSC and palliative care) into the model. Drug costs were calculated based on the bid-winning drug price from the China’s health industry data platform (https://www.yaozh.com/) (China’s health industry data platform, 2021). We modeled the model patients as having a body weight of 65 kg and a body surface area of 1.72 m^2^ (Lu et al., 2017), and then rounded each administration dosage to an integral multiple of the single-size vial to account for drug wastage (Sarfaty et al., 2021). Costs of AE management for each second- and third-line treatment were included as a frequency-weighted aggregate by multiplying the frequency of AEs reported in the clinical trials by the corresponding AEs management cost estimated using data derived from local hospitals (Lu et al., 2021a; Zhou et al., 2021). The model considered all observed grade III/IV AEs and were detailed in Supplementary Table S4. The costs of routine follow-up, subsequent anticancer therapy, BSC and palliative care were derived from previous literature (Liu et al., 2021b).

QALYs were computed as a discounted sum of Chinese-specific health state utilities for advanced NSCLC within the model runtime (Shen et al., 2018). Utility decrement caused by common grade III/IV AEs during second- and third-line treatment was also considered in the model (Nafees et al., 2017). The statistical method used for the utility decrement is similar to the method used for AE costs (Supplementary Table S4).

### Statistical analysis

Deterministic sensitivity analysis (DSA) and probabilistic sensitivity analyses (PSA) were carried out to test the robustness of our cost-effectiveness model. Multiple DSAs were performed on individual parameters that varied within the ranges listed in Table 1 to determine their impact on the results. The variable range of parameters was set to their 95% CIs (such as utilities and HRs), plus or minus 50% of the baseline values (such as costs) or 0–8% recommended by the guidelines (such as discount) (Chinese Pharmaceutical Association, 2020). PSA was performed on multiple parameters randomly sampled from the distribution listed in Table 1 to assess the uncertainty in model inputs on affecting the model outputs. During DSA, 1,000 Monte Carlo simulations were used to generate 1,000 ICER estimates for tislelizumab versus docetaxel or nivolumab**.**

In addition, considering the disparity in OS benefits of tisleizumab between the male and female subgroups, we performed subgroups analysis using the sex-special HR of OS reported in the corresponding clinical trials (Chinese Pharmaceutical Association, 2020; Lu et al., 2021a; Zhou et al., 2021).

## Results

### Base-case analysis

Compared with docetaxel, tislelizumab provided an additional 0.33 QALYs (1.37 vs. 1.04 QALYs) at an incremental cost of $9,286 ($23,646 vs. $14,360) for Chinese patients with driver-negative advanced or metastatic NSCLC, resulting in an ICER of $27,959/QALY under the WTP threshold of $35,663/QALY used in the model (Table 2). Compared with nivolumab, tislelizumab was associated with a lower cost ($23,646 vs. $59,447) and higher QALYs (1.37 vs. 1.20 QALYs), resulting in its dominance of nivolumab (Table 2).

**TABLE 2:** Summary of simulation results.

Base-caseact analysis	Cost, $	QALYs	Cost, $	Incremental	ICER,$/QALY
				QALYs	
Docetaxel	14,360	1.04			
Tislelizumab	23,646	1.37	9,286	0.33	27,959 (cost-effective)
Nivolumab	59,447	1.20	35,801	-0.18	Dominated
Subgroups analysis (male)					
Docetaxel	14,326	1.04			
Tislelizumab	25,131	1.48	10,805	0.44	24,448 (cost-effective)
Nivolumab	59,901	1.23	34,770	-0.26	Dominated
Subgroups analysis (female)					
Docetaxel	14,326	1.04			
Tislelizumab	20,211	1.12	5,885	0.07	80,683 (not cost-effective)
Nivolumab	59,660	1.21	39,449	0.09	428,246 (not cost-effective)

QALY, quality-adjusted life-years; ICER, incremental cost-effectiveness ratios.

### Sensitivity analysis

**FIGURE 2:**
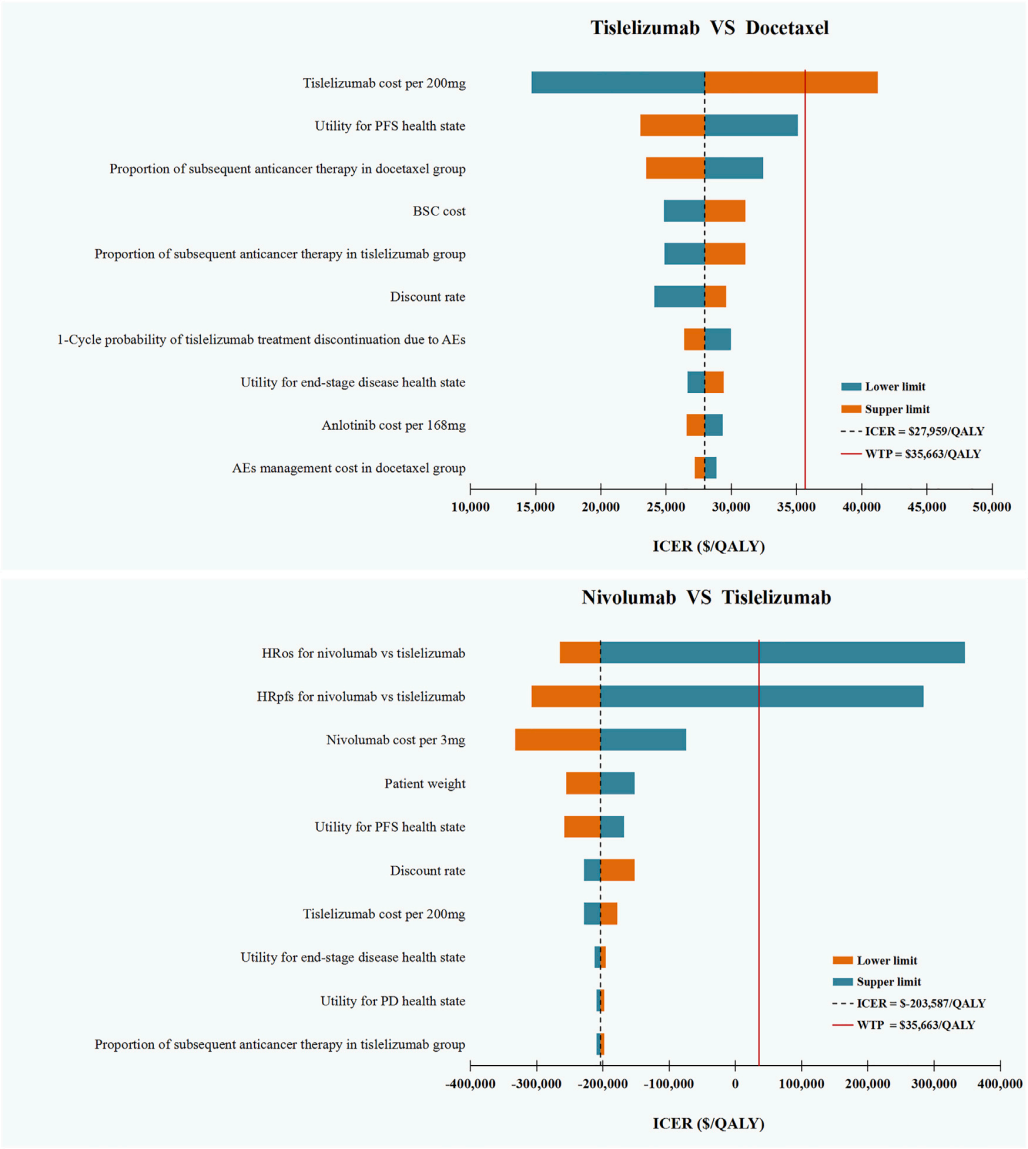
Deterministic Sensitivity Analysis. ICER, incremental cost-effectiveness ratios; QALY, quality-adjusted life-years; OS, overall survival; PFS, progression-free survival; HR, hazard ratio; PD, progressive disease; BSC, best supportive care; AEs: adverse events.

The DSA results of tislelizumab vs. docetaxel showed that tislelizumab cost per 200 mg was the only parameter that can shift the cost-effective strategy from tislelizumab to docetaxel when assuming a WTP threshold of $35,663/QALY. The variations in other parameters did not substantially alter our main results. The DSA results of tislelizumab versus nivolumab suggested that the HRs were the most influential parameter affecting our model. Variations in other parameters within reasonable ranges resulted in negative ICERs, which suggested that nivolumab was a cost-ineffective strategy compared with tislelizumab. The top 10 parameters relevant to ICERs are shown in Figure 2.

The PSA on the probability of tislelizumab to be cost-effective compared with the docetaxel and nivolumab revealed that, at a WTP threshold of $35,663/QALY, tislelizumab versus docetaxel was cost-effective in 42.1% of the 1,000 iterations and dominant in 12.0% (Supplementary Figure S1); tislelizumab versus nivolumab was cost-effective in 95.6% of the 1,000 iterations and dominant in 87.3%.

### Subgroup analysis

In the male subgroups, tislelizumab remained cost-effective than docetaxel with an ICER ($24,448/QALY) below the WTP threshold used in the model, and dominated nivolumab (Table 2). However, in the female subgroups, tislelizumab was not cost-effectiveness than docetaxel with an ICER ($80,683/QALY) far above the WTP threshold used in the model, but it is still preferable to nivolumab.

## Discussion

Domestic PD-1inhibitor has emerged as a promising treatment for stand-of-care in the management of driver-negative advanced or metastatic NSCLC in China (Guidelines Working Committee of Chinese society of Clinical Oncology, 2021). Although increasing clinical trials have confirmed the great clinical efficacy and favorable safety of domestic PD-1inhibitors (Wang et al., 2020; Lu et al., 2021b; Zhou et al., 2021), there are still limited pharmacoeconomic evidence on the cost-effectiveness of domestic PD-1inhibitors. This study uniquely demonstrates the cost-effectiveness advantage of domestic PD-1inhibitor tislelizumab immunotherapy compared with conventional docetaxel chemotherapy and imported PD-1inhibitor nivolumab immunotherapy in the treatment of driver-negative advanced or metastatic NSCLC beyond the first-line setting from the perspective of Chinese health care system.

Our current results, combined with the findings from our previous cost-effectiveness studies (Liu et al., 2021a; Liu et al., 2021b), have important implications on the appropriateness of treatment widespread use by considering the impact of costs anticancer drugs on treatment decision-making in routine clinical practice. At the patient level, costly anticancer drug may increase patients’ risks of substantial debt or bankruptcy, thus making the anticancer drugs less affordable (Shi et al., 2016). At the socioeconomic level, China has more than one-third newly diagnosed lung cancer cases worldwide (Cao et al., 2021; Sung et al., 2021). The huge patient population makes the use of expensive anticancer drugs imposing a great economic burden on Chinese healthcare system (Xu et al., 2022). To reduce the financial burden brought by the use of anticancer drugs, the Chinese government has implemented a series of “combination boxing of health-care reform” in recent years, including supporting domestic research and development of anticancer drugs (Li et al., 2021), negotiating with suppliers to reduce the anticancer drugs price (Tang et al., 2020), and adding anticancer drugs into the National Reimbursement Drug list (NRDL) to realize government-patient copayment (Central People’s Government of the People’s Republic of China, 2021b). Tislelizumab evaluated in this study is a domestic PD-1inhibitor, which is likely to attain support from Chinese medical reform policies in the future. Against this background, we can expect that the cost-effectiveness of tislelizumab in Chinese NSCLC patients will be further improved.

Whether domestic anticancer drugs can provide similar or greater therapeutic efficacy than the imported anticancer drugs remains unanswered. In the absence of head-to-head clinical trials, we conducted an indirect cost-effectiveness comparison through network meta-analysis for domestic PD-1inhibitor tislelizumab immunotherapy and imported PD-1inhibitor nivolumab immunotherapy in the second- or third-line treatment of Chinese patients with driver-negative advanced or metastatic NSCLC. Results from this economic evaluation showed that compared with nivolumab, treatment with tislelizumab enables Chinese NSCLC patients to achieve a prolonged survival at a lower total cost. This finding added evidence for the use of tislelizumab among Chinese patients with driver-negative advanced or metastatic NSCLC beyond first-line setting. However, sensitivity analyses regarding the uncertainty in model parameters found that HRs of nivolumab relative to tislelizumab have the potential to change our findings. Therefore, when more mature clinical data are available making a direct comparison possible, our results warrant a further validation.

The emergence of domestic anticancer drugs not only caters to the growing demand for cancer treatment in China, but also panders to the challenges in the era of “Universal Medical Insurance System”. To alleviate the catastrophic medical expenditure (commonly known as “kan-bing-nan”, “kan-bing-gui” in Chinese), China launched a major health-care reform in 2019 and pledged to establish a basic medical insurance system covering all citizens by 2020 (Yip et al., 2019). As a result, the Chinese government has to invest massive funding into the health-care sector: from 2009 to 2020, government health expenditure on health care has quintupled from $52.6 billion to $331.7 billion (31). At present, how to effectively save and reasonably use the medical insurance fund has become the key to maintaining the sustainable development of Chinese Universal Medical Insurance System. As it is well-known that the market price of domestic anticancer drugs is generally much lower than that of imported products (Li et al., 2021), their widespread use in cancer treatment can greatly reduce the financial pressure of medical insurance fund.

To our knowledge, this is the first cost-effectiveness study to evaluate domestic PD-1inhibitor in the second- or third-line treatment for driver-negative advanced or metastatic NSCLC in Chinese patients. In addition, this study is also the first to compared domestic PD-1inhibitor with imported PD-1inhibitor in this setting. Our findings contributed to the existing evidence base that supports the use of domestic anticancer drugs as cost-effective treatments for cancers and have important implications for Chinese government to balance the limited expenditure of medical insurance fund and the growing demand for cancer treatment.

This study has several limitations. First, the study evaluated the cost-effectiveness of a novel domestic PD-1inhibitor tislelizumab in second- or third-line treatment of Chinese patients with driver-negative advanced or metastatic NSCLC, which was only recently reported in the RATIONALE 303 clinical trial and is still being assessed in an ongoing trial. Second, we used health state utilities reported in previous literature to inform model because quality-of-life data have not been published along with the main results of the RATIONALE 303 clinical trial to date, although literature-based utilities were specific to Chinese NSCLC patients (Shen et al., 2018). Third, the proportion of patients receiving subsequent anticancer therapy after progressing on tislelizumab, nivolumab and docetaxel treatment were derived from clinical trials (Lu et al., 2021a; Zhou et al., 2021), which may not fully reflect the real-world clinical practice. However, we varied these parameters by 50% around the baseline values in sensitivity analyses and found our results were robust. Fourth, there is an uncertainty in the post-trial outcomes for patients, although the long-term survival was inferred from KM curves using validated extrapolation techniques. Sixth, due to the lack of head-to-head clinical trials comparing these 3 drugs (tislelizumab, nivolumab and docetaxel), the HRs generated from a NMA was employed to enable this indirect cost-effectiveness comparison. However, as the treatment effect may differ between males and females and the ratios of males to females are different across trials, the indirect comparison may introduce some biases.

In conclusion, domestic PD-1inhibitor tislelizumab immunotherapy represents a cost-effective treatment strategy compared with conventional docetaxel chemotherapy and imported PD-1inhibitor nivolumab immunotherapy in the treatment of driver-negative advanced or metastatic NSCLC beyond the first-line setting from the perspective of Chinese health care system. In the era of “Universal Medical Insurance System”, the rational use of domestic anticancer drugs guided by cost-benefit evidence would be an effective means to balance the limited expenditure of medical insurance fund and the growing demand for cancer treatments.

## Data Availability

The original contributions presented in the study are included in the article/Supplementary Material, further inquiries can be directed to the corresponding author.
