# Supplementary figures and images for: Cost-effectiveness analysis of tislelizumab, nivolumab and docetaxel as second- and third-line for advanced or metastatic non-small cell lung cancer in China
Source: Front Pharmacol. 2022 Aug 25;13:880280. doi: 10.3389/fphar.2022.880280 (PMC9453816; doi:10.3389/fphar.2022.880280)

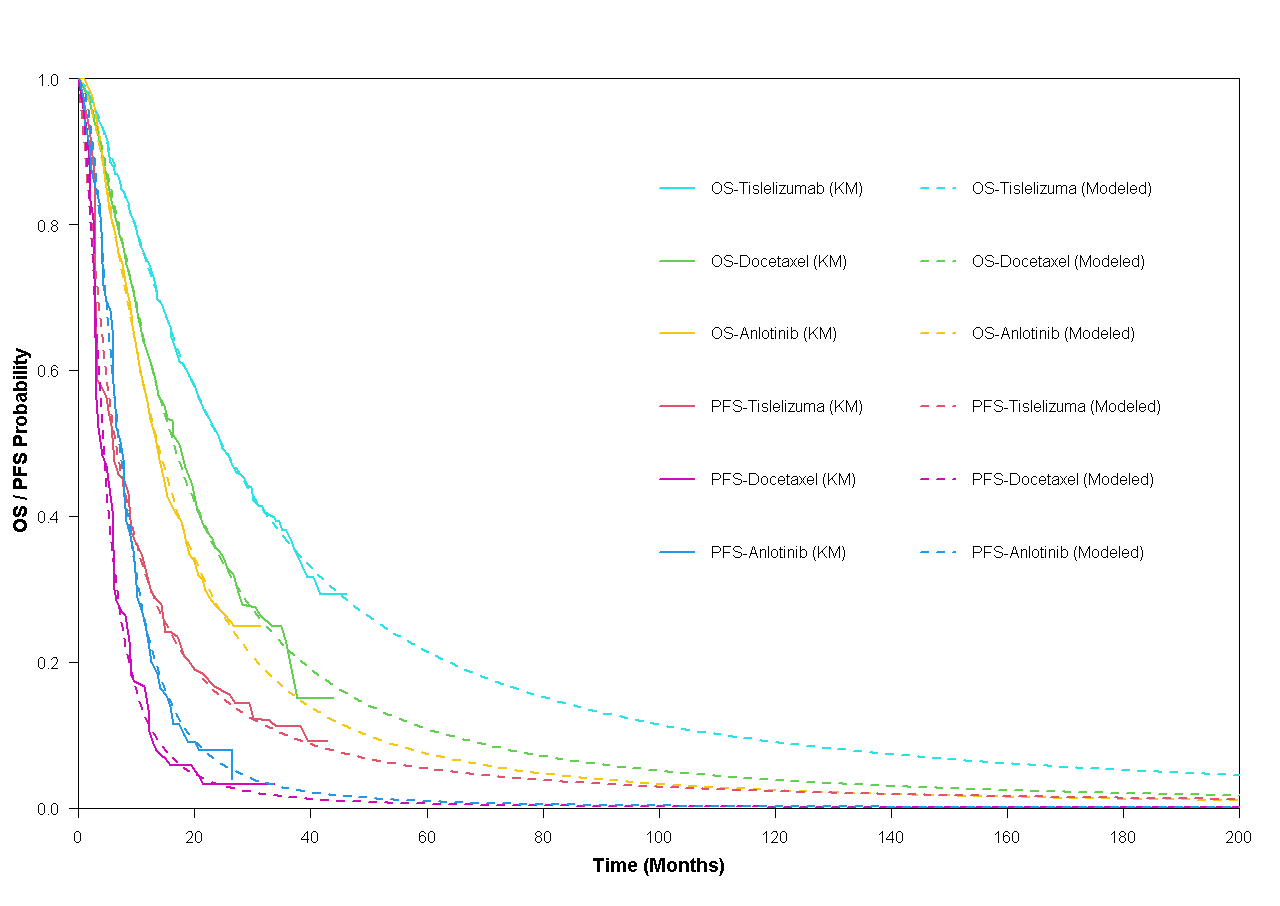

Supplement: Supplementary file 1 [file Image1.TIFF]

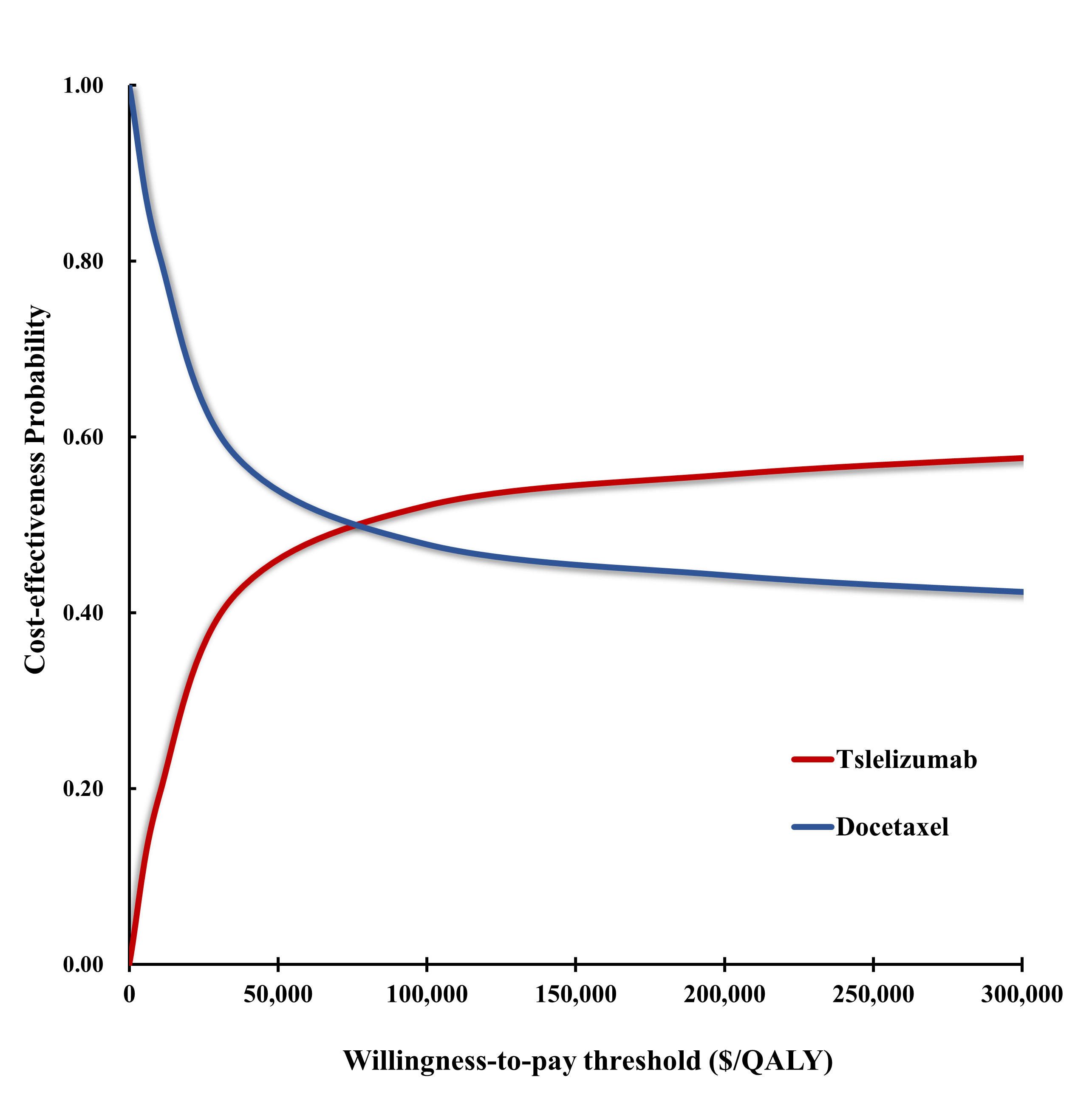

Supplement: Supplementary file 3 [file Image2.TIF]
