# Supplementary material for: Cost-effectiveness analysis of tislelizumab, nivolumab and docetaxel as second- and third-line for advanced or metastatic non-small cell lung cancer in China
Source: Front Pharmacol. 2022 Aug 25;13:880280. doi: 10.3389/fphar.2022.880280 (PMC9453816; doi:10.3389/fphar.2022.880280)
Supplement: Supplementary file 2 [file Table1.DOCX]

Table S1. Treatment regimens used in the model.

| **Regimens** | **Dosage** | **Administration** | **Proportion of** **subsequent anticancer therapy** |
| --- | --- | --- | --- |
| Second- or third-line  Tislelizumab | 200mg | On day 1 of every 3 weeks | 49.7% ^a^ |
| Second- or third-line  Docetaxel | 75mg/m^2^ | On day 1 of every 3 weeks | 45.0% ^a^ |
| Second- or third-line  Nivolumab | 3 mg/kg | On day 1 of every 2 weeks | 62.6% ^b^ |
| Third- or further-line  Anlotinib | 12 mg/d | Daily for the first 2 weeks of every 3 weeks | / |

*^a^* *The proportions of patients receiving subsequent anticancer therapy were collected from the RATIONALE 303* *clinical trial.*

*^b^* *The proportions of patients receiving subsequent anticancer therapy were collected from the CheckMate 078 clinical trial.*
