# Supplementary material for: Cost-effectiveness analysis of tislelizumab, nivolumab and docetaxel as second- and third-line for advanced or metastatic non-small cell lung cancer in China
Source: Front Pharmacol. 2022 Aug 25;13:880280. doi: 10.3389/fphar.2022.880280 (PMC9453816; doi:10.3389/fphar.2022.880280)
Supplement: Supplementary file 4 [file Table2.DOCX]

Table S2. AIC and BIC statistics for alternate parametric distributions.

| **Parametric Distribution** | **Second- or Third-line Tislelizumab** | | | | **Second- or Third-line Docetaxel** | | | | **Third- or Further-line Anlotinib** | | | |
| --- | --- | --- | --- | --- | --- | --- | --- | --- | --- | --- | --- | --- |
|  | **OS data** | | **PFS data** | | **OS data** | | **PFS data** | | **OS data** | | **PFS data** | |
|  | **AIC** | **BIC** | **AIC** | **BIC** | **AIC** | **BIC** | **AIC** | **BIC** | **AIC** | **BIC** | **AIC** | **BIC** |
| Exponential | -396 | -392 | -108 | -105 | -206 | -202 | -113 | -110 | -151 | -147 | -121 | -118 |
| Weibull | -483 | -476 | -123 | -118 | -255 | -250 | -130 | -125 | -187 | -182 | -184 | -178 |
| Lognormal | -538 | -530 | -141 | -135 | -258 | -253 | -157 | -152 | -239 | -233 | -203 | -197 |
| Loglogistic | -579 | -572 | -148 | -143 | -263 | -257 | -157 | -152 | -246 | -241 | -216 | -210 |

*OS, overall survival; PFS, progression-free survival; AIC, Akaike information criterion; BIC, Bayesian information criterion.*
