# Supplementary material for: Cost-effectiveness analysis of tislelizumab, nivolumab and docetaxel as second- and third-line for advanced or metastatic non-small cell lung cancer in China
Source: Front Pharmacol. 2022 Aug 25;13:880280. doi: 10.3389/fphar.2022.880280 (PMC9453816; doi:10.3389/fphar.2022.880280)
Supplement: Supplementary file 5 [file Table3.DOCX]

Table S3. Proportion and probability of second- or third-line treatment discontinuation due to AEs.

| **Regimen** | **Number of as-treated patients** | **Number of patients experiencing event** | **Median OS**  **(months)** | **Proportion** | **Instantaneous rate** | **1-cyle probabilities** |
| --- | --- | --- | --- | --- | --- | --- |
| **Tislelizumab** | 534 | 56 | 17.2 | 0.104869 | 0.004509 | 0.004499 |
| **Docetaxel** | 258 | 32 | 11.9 | 0.124031 | 0.007790 | 0.007759 |
| **Nivolumab** | 337 | 19 | 11.9 | 0.056380 | 0.003414 | 0.003408 |

*OS, overall survival; AEs, adverse events.*

*The following formula was applied to convert the proportion of patient experiencing AEs-related treatment discontinuation event in clinical trial period into a 1-cylce probability:* $P=1-exp(-rt)$*, where p indicates the probability, r is the instantaneous rate and t is the time period.*
