# Supplementary material for: Cost-effectiveness analysis of tislelizumab, nivolumab and docetaxel as second- and third-line for advanced or metastatic non-small cell lung cancer in China
Source: Front Pharmacol. 2022 Aug 25;13:880280. doi: 10.3389/fphar.2022.880280 (PMC9453816; doi:10.3389/fphar.2022.880280)
Supplement: Supplementary file 6 [file Table4.DOCX]

Table S4. Proportion, costs and disutility of grade III/IV AEs considered in the model.

| AEs | Proportion (%) | | | Cost per event($)^a^ | Disutility |
| --- | --- | --- | --- | --- | --- |
|  | Tislelizumab | Docetaxel | Nivolumab |  |  |
| Anemia | 3.4% | 6.2% | 0.3% | 2150.12 | /^c^ |
| ALT increased | 0.7% | 0.0% | 0.0% | 292.59 | /^c^ |
| Cough | 0.9% | 0.4% | 0.0% | 90.88 | /^c^ |
| AST increased | 0.9% | 0.4% | 0.0% | 292.59 | /^c^ |
| Decreased appetite | 0.9% | 1.2% | 0.0% | /^b^ | /^c^ |
| Weight decreased | 0.7% | 0.0% | 0.0% | /^b^ | /^c^ |
| Hypoalbuminemia | 0.0% | 0.4% | 0.0% | 585.94 | /^c^ |
| Asthenia/Fatigue | 1.1% | 5.4% | 0.9% | /^b^ | 0.07 |
| Nausea | 0.0% | 0.4% | 0.0% | 17.22 | 0.12 |
| WBC count decreased | 0.2% | 18.2% | 0.3% | 802.35 | /^c^ |
| Neutrophil count decreased | 0.6% | 27.5% | 0.0% | 628.91 | /^c^ |
| Leukopenia | 0.2% | 15.9% | 0.3% | 802.35 | /^c^ |
| Neutropenia | 0.6% | 27.9% | 0.3% | 628.91 | 0.20 |
| Alopecia | 0.0% | 0.8% | 0.0% | /^b^ | 0.06 |
| Rash | 0.0% | 0.0% | 0.9% | 26.79 | 0.10 |
| Estimated AEs Costs and disutility | | | | | |
| AEs cost for second- or third-line tislelizumab, $ | | | | 89.36 |  |
| AEs cost for second- or third-line docetaxel, $ | | | | 1212.99 |  |
| AEs cost for second- or third-line nivolumab, $ | | | | 13.39 |  |
| AEs disutility for second- or third-line tislelizumab | | | | | 0.002 |
| AEs disutility for second- or third-line docetaxel | | | | | 0.061 |
| AEs disutility for second- or third-line nivolumab | | | | | 0.002 |

*AEs, adverse events; ALT, alanine aminotransferas; AST, aspartate aminotransferase; WBC, white blood cell.*

*^a^The AEs management cost used in this analysis were estimated from local hospitals.*

*^b^Based on the local oncologists’ opinions and clinical practices, these AEs do not require additional treatment.*

^c^*The disutility regarding these AEs were not reported.*
